# Constrains for seeking post-abortion care among adolescents and young women in Guangzhou, China: a cross-sectional study

**DOI:** 10.1186/s12913-021-06263-0

**Published:** 2021-05-28

**Authors:** Yiding Wang, Jinzhi Liu, Ribo Xiong, Yan Liu

**Affiliations:** 1 Department of gynecology &obstetrics, The Third Affiliated Hospital of Southern Medical University, Guangzhou, China; 2 Department of rehabilitation, Nanhai Hospital, Southern Medical University, Foshan, China

**Keywords:** Abortion, Post-abortion care, Utilization, Stigma, Adolescents, Young women, China

## Abstract

**Background:**

In China, post-abortion care (PAC) services mainly focus on married couples, such that adolescents and unmarried young womenhave limited access to those services for contraception counseling. The provision of youth-friendly PAC services in public hospitals is a new concept in China. This study examined the magnitude of PAC services utilization as well as factors influencing it’s uptake among adolescents and young women in Guangzhou, China.

**Methods:**

A cross-sectional study was performed from 1st March 2020 to 30th September 2020 using anonymous self-administered questionnaire among 688 women aged 15–24 years in Tianhe district, Guangzhou. The Multivariate logistic regression was used to determine factors that were significantly associated with the uptake of PAC services.

**Results:**

The magnitude of PAC services utilization was 35.9% among adolescents and young women in Guangzhou, China. Students were 69.0% significantly less likely to use PAC services compared to women who had no job. Immigrants were 59.0% significantly less likely to use PAC services than their native counterparts. Women who had a feeling of stigma were 70.0% significantly less likely to use PAC services compared to those who did not feel stigmatized.

**Conclusions:**

The study highlights the need to strengthen youth-friendly PAC services provision, and emphasizes the importance of education about both family planning and abortion services among disadvantaged sub-groups of women in the study setting.

## Background

High abortion rates among sexually active youth in China are a serious public health concern central to government policy on tackling reproductive health [1]. The large proportion of unintended pregnancies and subsequent induced abortions in this age group is driven, in their limited knowledge about reproduction and contraception, as well as a lack of access to family planning services [2]. Data published by China’s National Health and Family Planning Commission indicates that almost half of the abortions were conducted on women below the age of 25 [1]. It’s difficult to fully ascertain the number of adolescents and youth nationwide who have experienced abortion procedures before the age of 25 even though abortion is legal and available in China. Evidence also suggests that pregnancy termination is likely to cause long-lasting physical and psycho-social reactions for women who seek them [3]. This is especially true for the relatively mentally immature group of younger youth aged 15–18 years, who do not always possess the understanding, skills or support to deal with clinical and social consequences.

To address the complications related to abortions, post-abortion care (PAC) has been introduced to break the cycle of unwanted pregnancy and improve women’s sexual and reproductive health. Standard PAC includes contraceptive counseling and contraceptive methods provision; emergency care; human immunodeficiency virus counseling; and community empowerment [4]. Prior studies in China have demonstrated that PAC utilization was directly related to marital status, knowledge of fertility return after abortion, decision maker on having a child, and service providers’ perspectives [5, 6]. However, most of the participants recruited in these studies were women without any restrictions or young unmarried women. Further, PAC services in China mainly focus on married couples, which means young girls have limited access to these services. Youth-friendly family planning counseling is not explicitly fostered in the current context or included in standard counseling approaches. Although PAC within public abortion clinics is provided free of charge, the implication is that young people encounter more obstacles towards acquiring PAC compared with older adults, resulting in the low rate of contraception use and subsequent repeat abortions. How the specific context affects the implementation of PAC and what efforts are needed to address the barriers to PAC services among this population continuously attract researcher’s interests.

The World Health Organization identifies differences in the needs of young people (from 15 to 24 years old) as PAC service clients and recommends specific guidelines that maximize protection against unintended pregnancy [4]. China offers an interesting context for understanding PAC utilization and its associated factors among adolescents and young women. In 2017, the Chinese government launched a Medium-and Long-Term Development Program for young people (14–35 years) to strengthen the popularization of sexual knowledge and implement sexual health education [5]. Guangzhou, where this study was conducted, was one of the pilot cities where youth-friendly family planning was included in standard counseling to address young people’s specific concerns and needs. A non-judgemental stance in counseling was also developed to discuss their ambivalent attitudes towards sexuality. Yet the extent to which PAC services are utilized and it’s contributing factors in such settings are not well-understood. In addition, in the past decade, China has experienced dramatic social changes with young people rushing to costal developed regions such as Shanghai and Guangzhou to seek better livelihood. Previous studies have reported immigrant women were less likely to use PAC services [6, 7]. However, little is known on the factors determining their seeking PAC services, especially young immigrant women who may delay the request for termination and subsequent family planning counseling to forestall stigmatization. This is a limitation given the changing demographics of young abortion patients in this area. This study aims to assess PAC utilization among adolescents and young women, and explore the potential factors influencing its uptake in Guangzhou, China.

## Methods

Guangzhou is an economically developed city in south China and is one of the largest metropolitans. Tianhe district, where this study was conducted, was the site where youth-friendly PAC services were pilotly provided in public hospitals. This district has the largest migrant population and college students in Guangzhou.

This was a cross-sectional study conducted in Tianhe district between March and September in 2020. We recruited women 1) at an age between 15 and 24 years; 2) having experienced at least once induced abortions; 3) providing informed consent. Women who had a history of psychiatric disorder or returned incomplete questionnaires were excluded.

Based on sample size estimation, 568 women were needed where effect size was set at 0.5, α = 0.05 referring the study of Tang et al. [7] Considering a non-response rate of 20.0%, we aimed to recruit 682 women. A total of 813 eligible women were invited to participate; 122 women refused to be enrolled in and 3 women returned incomplete questionnaires, resulting 688 women. This study included 688 adolescents and young women allowing detection of significant differences with a power of 0.85 calculated by Gpower software.

Four public hospitals that provided youth-friendly PAC pilotly were included in the study. The number of participants from each hospital was determined based on population proportion to size. The number of participants was 235, 76, 201 and 176 from the first, second, third and fourth hospital respectively.

An anonymous self-administered questionnaire was used and completed by participants. The questionnaire was comprised of four sections:(1) general socio-demographic information, such as age, education, occupation, marital status, average monthly income and migration status; (2) reproductive history, such as parity, previous induced abortion and number of living children; (3) contraceptive practices, such as contraceptive knowledge and contraceptive use during the six months preceding survey; (4) social factors, such as a feeling of stigma, other people’s (e.g partner, husband, parents) attitudes towards contraceptive use, person who determine the utilization of PAC services, fear that contraceptives are harmful and negative attitudes towards PAC services.

All the participants were assured confidentiality of their identity and responses before the survey. Small gifts (sanitary towel) were given to them when they returned the questionnaire. Data was collected by four female nurses/midwives with field research experience who were trained by the principal investigator for two days.

Ethics approval was obtained from Ethical Committee of Southern Medical University. Participants were asked to sign a consent form prior to the study. For participants under 18 years old, written informed consent was obtained from her parent or guardian.

Data was analyzed using SPSS16.0. Cross-tabulations with Chi-square test were used evaluate the significance of differences between abortion patients who utilized PAC services and those who did not. Then the independent variables that were significantly associated (*P* < 0.05) with the uptake of PAC services were considered as possible contributing factors and entered into a multivariate logistic regression model. Odds ratios (ORs) with 95% confidence intervals (95% CI) were calculated to measure the strength of association. A *P* value<0.05 was considered significant in the analysis.

## Results

Of the 688 abortion patients who were enrolled in this study, 247 utilized PAC services, thus resulting a magnitude of 35.9%.

86.3% of the participants were aged between 19 and 24 years and less than one fifth (13.7%) were adolescents. A majority (44.9%) of the participants had college level education or more. More than half (69.0%) of the participants were unmarried. Regarding occupation, 46.9% of the participants were students. The highest proportion (46.2%) of participants earned between 1000 and 4000 Chinese yuan per month, followed by those who earned less than 1000 Chinese yuan (29.7%). Distribution by migration status shows that 64.8% of the participants were immigrants. (Table 1)

**Table 1:** Socio-demographic features of abortion patients

Variables	Frequency	Percentage (%)
Age		
15 ~ 18	94	13.7
19 ~ 24	594	86.3
Marital status		
Married	213	69.0
Not married	475	31.0
Level of education		
Junior high school or less	178	25.9
Senior high school	201	29.2
College or more	309	44.9
Occupation		
Jobless	45	6.5
Student	323	46.9
Technician	27	3.9
Clerical staff/worker	159	23.1
Self-employed	134	19.5
Average monthly income		
<1000 CNY	204	29.7
1000 ~ 4000CNY	318	46.2
>4000 CNY	166	24.1
Migration status		
Non-migrant	242	35.2
Immigrant	446	64.8

CNY: Chinese Yuan

Immigrant: individuals who do not have permanent resident certificate and be living over a month in Guangzhou

The proportion of PAC utilization was significantly lower among students (29.1%) than among clerical staff/worker (40.0%), technician (44.4%) and jobless (73.3%). The proportion of immigrants that used the services was almost two times lower than that of non-immigrants (26.0 and 54.1% respectively). (Table 2). In addition, the proportion that used the services was significantly higher among women who did not feel stigmatized (70.2%)than among those who had a feeling of stigma (21.1%) or those who were uncertain (12.2%). (Table 3). However, there were no statistically significant variations in the proportions of PAC services utilization by other factors considered. (Tables 2, 4, and 5).

**Table 2:** Variations in the use of PAC services by socio-demographic features

Characteristics	Total	Use of PAC services		χ^**2**^	***P***
		Frequency	Percentage(%)		
Marital status				0.668	0.363
Married	213	79	37.1		
Not married	475	168	35.3		
Level of education				1.614	0.446
Junior high school or less	178	57	32.0		
Senior high school	201	76	37.8		
College or more	309	114	36.9		
Occupation				35.549	0.000
Jobless	45	33	73.3		
Student	323	94	29.1		
Technician	27	12	44.4		
Clerical staff/worker	159	62	40.0		
Average monthly income				0.455	0.797
<1000 CNY	204	75	36.8		
1000 ~ 4000CNY	318	110	34.6		
>4000 CNY	166	62	37.3		
Migration status				0.000	0.000
Non-migrant	242	131	54.1		
Migrant	446	116	26.0

**Table 3:** Variations in the use of PAC services by reproductive history

Reproductive history	Total	Use of PAC services		χ^**2**^	***P***
		Frequency	Percentage(%)		
Parity				0.639	0.727
0	205	69	33.7		
1	233	86	36.9		
≥ 2	250	92	36.8		
Previous induced abortion				0.937	0.490
Yes	350	125	35.7		
No	338	122	36.1		
Number of living children				0.465	0.254
0	419	155	37.0		
1	269	92	34.2

**Table 4:** Variations in the use of PAC services by contraceptive practices

Contraceptive practices	Total	PAC services utilization		χ^**2**^	***P***
		Frequency	Percentage(%)		
Knowledge on how soon fertility returns and could get pregnant again				1.104	0.576
Within 10 ~ 14 days	212	80	37.7		
After 3 ~ 4 weeks	216	80	37.0		
Don’t know	260	87	33.5		
Any uptake of contraception				1.098	0.578
Yes	283	96	33.9		
No	341	129	37.8		
Missing	64	22	34.3		
Contraceptive use (multiple responses allowed)					
None	259	92	35.5	0.935	0.469
Traditional methods	138	51	36.9	0.767	0.423
Oral contraceptives	282	95	33.7	0.333	0.177
Male condoms	357	129	36.1	0.937	0.479

Traditional methods: rhythm, lactational amenorrhea, and withdrawal

**Table 5:** Variations in the use of PAC services by social factors

Variables	Total	PAC services utilization		χ2	P
		Frequency	Percentage(%)		
Having a feeling of stigma				177.1	0.000
Yes	345	73	21.1		
No	228	160	70.2		
Uncertain	115	14	12.2		
Spouses’/partners’ attitudes on contraceptive use				5.012	0.082
Approve	260	107	41.2		
Disapprove	244	80	32.8		
Uncertain	184	60	32.6		
Person responsible for PAC utilization				0.096	0.953
Spouse/partner/Parents	235	86	36.6		
Women herself	223	80	35.9		
Both	230	81	35.2		
Concerns about side-effects of contraceptives				0.102	0.751
Yes	351	124	35.3		
No	337	123	36.4		
Negative attitudes towards PAC services				0.557	0.757
Yes	240	82	34.2		
No	227	85	37.4		
Uncertain	221	80	36.2		

Table 6 showed the factors associated with PAC utilization among adolescents and young women in the study site. Students were 69% significantly less likely to use PAC services compared to women who had no job (OR = 0.308, 95%CI = 0.129 ~ 0.453). Similarly, immigrant women were 59% less likely to receive PAC services compared to native women (OR = 0.409, 95%CI = 0.137 ~ 0.620). The likelihood of using PAC services was also varied by whether women had a feeling of stigma. Women who had a feeling of stigma were 70% significantly less likely to use PAC services compared to those who did not feel stigmatized (OR = 0.296, 95%CI = 0.104 ~ 0.378).

**Table 6:** Odds ratios from multivariate logistic regression examining factors associated with PAC utilization among adolescents and young women in Guangzhou, China

Covariates	***OR***	***SE***	***95% CI***	***P***
Occupation				
Jobless	–	–	–	–
Student	0.308	0.231	0.129 ~ 0.453	0.000
Technician	0.621	0.567	0.289 ~ 0.961	0.551
Clerical staff/worker	0.396	0.127	0.059 ~ 0.326	0.067
Self-employed	0.215	0.313	0.116 ~ 0.627	0.078
Migration status				
Non-migrant	–	–	–	–
Migrant	0.409	0.094	0.137 ~ 0.620	0.000
Having a feeling of stigma				
Yes	0.296	0.225	0.104 ~ 0.378	0.000
No	–	–	–	–
Uncertain	0.608	0.129	0.009 ~ 0.413	0.068

## Discussion

We conducted a study aimed at determining the proportion of adolescents and young women who used PAC services and possible factors associated with it’s uptake. To our knowledge, this was one of the first studies with respect to the utilization of PAC services among adolescents and young women. Almost all existing studies in China were conducted among women without restrictions or the young unmarried women.

In this study, a minority of the participants, 247 (35.9%), used PAC services before they leave the facility. Uptake of PAC services among adolescents and young women in the study setting was lower than the level observed among women without restrictions in the same region [6]. Lower rate of PAC services utilization could be due to two evident variations between other studies and the current one. First, the present data were obtained from adolescents and young women. Women are initiating their sexual maturity earlier than ever and becoming sexual active prior to marriage due to the dramatic social changes in China in the past decade [8]. However, there is a lack of education on sex and reproductive knowledge in the traditional education system of China, leading to misconceptions about contraception and family planning counseling, especially compared to their western counterparts [8]. In addition, misled by deceptive advertising claiming painless abortion, Chinese young women, especially adolescents, no longer take the matter seriously, as if abortion was safe, affordable and with no impact on work. Therefore, these factors have helped to discourage adolescents and young women from seeking PAC. Second, the current provision of PAC in China does not address young people’s concerns and needs. Young women, especially adolescents are often implicitly or explicitly blamed for their use of abortion or family planning counseling. Moreover, globally, providers in PAC services have demonstrated judgemental stance towards young people in counseling [9]. Specific attitudes and skills are not explicitly fostered. Such approaches have prevented them from seeking post-abortion counseling.

The findings of this study showed that being a student was significantly less likely to use PAC services compared to their jobless counterparts. This was consistent with the concept that women’s empowerment and autonomy play a significant role in the process of seeking PAC [10]. Normally females in China aged 18 or below should still be receiving an education and the proportion of female students receiving college education is increasing. In comparison with their peers with formal or informal jobs, the majority of them are dependent on their parents for tuition and monthly pocket money. This finding proved some level of incompetency on the part of this group and their weak social support networks. Interestingly, in this case, we found that 92.8% of students were receiving college education. However, a higher level of education did not appear to enhance their possibility of seeking PAC, which was inconsistent with previous reports that women with higher educational level were more likely to seek PAC [11, 12]. The gap of reproductive and sexual education in college education system between China and other countries may partially explain this paradox. This outcome also implied that college students were already involved in sexual activities and were at risk of subsequent repeat induced abortions. Further research on class year of students (first year students vs. second year and higher class year students) and college major (social sciences and humanities vs. natural sciences) may be helpful to explore possible barriers to PAC services utilization. The finding suggests that local governments need to strengthen the popularization of sexual knowledge and promote sexual health education in universities. Furthermore, a substantial proportion (86.7%) of jobless women was married in this study, this may point to their good awareness of reproduction and contraception. Previous study has found that married women were more likely to utilize PAC compared to unmarried women [13]. This evidence, combined with the above explanations, is therefore likely to put students at a greater risk of not seeking PAC compared with jobless women, despite the financial constrains noted in both groups.

Our study echoes other studies that immigrant women were significantly less likely to use PAC services compared to their native counterparts [6]. Only a quarter received PAC services, suggesting limited or denial of access to family planning counseling. Immigrants in our study refer to the individuals who do not have permanent resident certificate and be living over a month in Guangzhou. They were reluctant to seek PAC probably due to their lacking of medical insurance in the host cities. Because the payment system of medical expenses is based on household registration system, immigrants have much less reimbursement than their natives. Although PAC is free of charge, contraceptives, particularly long acting reversible contraceptives are not free of charge. Unintended pregnancies will continue to occur due to their lack of counseling or non-use of contraceptives. Underutilization of PAC services among migrant women compared to native women suggests the need for policies targeting migrant women who often face unmet family planning needs. This could include the integration of migrant women into the welfare structures in their host cities.

Another key finding of this paper is that women who had a feeling of stigma were significantly less likely to use PAC services compared to those who did not have such a feeling. This is consistent with findings in other qualitative studies assessing women’s abortion experiences [14]. Women experiencing stigmatized abortion views may be misled by the perception that they are violating social norms, such as women’s expected role as mothers. This view may contribute to their delays in pursuing an abortion as well as seeking PAC. Additionally, young women, especially teenagers may refuse to be presented at PAC services in order to maintain secrecy to reduce the number of people who become aware that they had an abortion to forestall stigmatization. Efforts should be made to address community level attitudes and beliefs on abortion as well as reinforce the role of PAC in helping women better reproductive health. However, these interventions should be cautious of not further stigmatizing women. Further research is needed to explore the role of stigma-related barriers in determining the utilization of PAC services among adolescents and young women in China.

This study has some limitations. First, the generalizability of this study would be limited as we only conducted the study in public hospitals. We might have missed out the proportion of women who seek PAC in private clinics. Second, report bias may exist in the survey although we used anonymous self-administered questionnaire. Given the sensibility of the topic, adolescents and young women might provide responses they feel socially desirable. Third, we did not ask women to list all sources of stigma.

## Conclusion

Uptake of PAC services remains low among adolescents and young women in the study setting where the provision of youth-friendly services in public hospitals was being experimented. Underutilization of the services occurs among students, immigrants, and those who had a feeling of stigma. The findings suggest the need for policies and programs to not only strengthen the provision of youth-friendly services but also promote education about both family planning and abortion services among disadvantaged sub-groups of women.

## Data Availability

The datasets used and/or analyzed during the current study are available from the corresponding author on reasonable request.

## Abbreviation

PAC: Post-abortion care I
